# Association Between Cholangiocarcinoma and Proton Pump Inhibitors Use: A Nested Case-Control Study

**DOI:** 10.3389/fphar.2018.00718

**Published:** 2018-07-03

**Authors:** Yen-Chun Peng, Cheng-Li Lin, Wan-Yun Hsu, Wai-Keung Chow, Show-Wu Lee, Hong-Zen Yeh, Chia-Chang Chen, Chia-Hung Kao

**Affiliations:** ^1^Division of Gastroenterology, Department of Internal Medicine, Taichung Veterans General Hospital, Taichung, Taiwan; ^2^National Yang-Ming University, Taipei, Taiwan; ^3^Management Office for Health Data, China Medical University Hospital, Taichung, Taiwan; ^4^School of Medicine, College of Medicine, China Medical University, Taichung, Taiwan; ^5^Department of Nursing, Taichung Veterans General Hospital, Taichung, Taiwan; ^6^Division of Gastroenterology, Taichung Tsu-Chi Hospital, Taichung, Taiwan; ^7^Graduate Institute of Clinical Medical Science and School of Medicine, College of Medicine, China Medical University, Taichung, Taiwan; ^8^Department of Nuclear Medicine and PET Center, China Medical University Hospital, Taichung, Taiwan; ^9^Department of Bioinformatics and Medical Engineering, Asia University, Taichung, Taiwan

**Keywords:** cholangiocarcinoma, proton pump inhibitors, case-control study, defined daily dose, odds ratios

## Abstract

**Background:** The present study aimed to examine the odds of cholangiocarcinoma (CCA) in patients with proton pump inhibitors (PPIs) use.

**Methods**: A nested case-control study design was employed using data obtained from Taiwan's National Health Insurance Research Database. In total, 2,293 patients with confirmed diagnosis of CCA were identified and served as the CCA group. The CCA patients were propensity score-matched with 2,293 subjects without CCA who served as the control group. The cumulative defined daily dose (DDD) of PPIs was calculated based on the total supply in days and quantity of individual PPIs. Univariable and multivariate logistic regression models were used to determine the odds of CCA, and calculated odds ratios (ORs) and 95% confidence intervals (CI) were used to assess PPIs use and odds of CCA.

**Results:** The overall adjusted OR of PPIs use-associated CCA was 2.58 (95% CI 2.27, 2.93). The adjusted OR of CCA by cumulative DDD dose of PPIs and CCA was analyzed and revealed those odds of CCA are associated with all types of PPIs.

**Conclusions:** There were odds of intrahepatic and extrahepatic CCA among PPIs users. All PPIs use was associated with odds of CCA. Analyses of larger numbers of cases are needed to confirm these findings.

## Introduction

Proton pump inhibitors (PPIs) are widely used for gastric acid-related disorders because they are generally safe and effective (Fortinsky et al., 2015; Fock et al., 2016). The long-term use of PPIs is becoming an important issue with respect to safety and the most concerning adverse effects of PPIs are related to nutrition, drug interactions, infections, and bone metabolism (Yang and Metz, 2010). Neoplasia is an important concern with long-term use of PPIs, such as gastric cancer, pancreatic cancer, and peri-ampullary cancer (Poulsen et al., 2009; Yang and Metz, 2010; Bradley et al., 2012; Chien et al., 2016; Cheung et al., 2018).

PPIs also exhibit pleotropic effects including anti-cancer and anti-inflammatory effects such as anti-oxidant properties and immunomodulatory effects through their interactions with neutrophils, monocytes, endothelial, and epithelial cells *ex vivo* (Yoshida et al., 2000; Simon et al., 2006; Namazi and Jowkar, 2008). Epidemiological data PPIs are considered to possibly increase the odds of carcinogenesis, but data is still conflicting (Chien et al., 2016; Cheung et al., 2018). They have also been shown to exert chemo-preventive effects in some types of tumors (Morimura et al., 2008; Miyashita et al., 2013; Han et al., 2015). PPIs also have selective anti-cancer effects via apoptosis of tumor (Huang et al., 2013), and sensitization of cancer cells to chemotherapy and radiotherapy (Wang et al., 2015). The limited data suggest that the role of PPIs in carcinogenesis requires further investigation.

Cholangiocarcinoma (CCA) is the most common biliary tract malignancy, and the second most common cancer of liver malignancies. The globalincidence of cholangiocarcinoma iswide variable, ranging from highest in northeastThailand, with age-standardized incidence rates of approximately 100 per 100,000 individuals amongmen and 50 per 100,000 individuals among women, and inthe West, range between 0.5 and 2.0 per 100,000 individuals (Banales et al., 2016). The prognosis of CCA isusually considered dismal. Late diagnosis compromises the effective therapeutic options, surgical resection or liver transplantation and chemotherapies are usually considered to be palliative.The significant cancer burden, and high mortality result in a health problem that warrants considerable attention (Banales et al., 2016; Treeprasertsuk et al., 2017) CCA tends to develop on the background of inflammation and cholestasis. In addition to known established associated factors, novel possible associated factors (i.e., obesity, hepatitis B virus, hepatitis C virus) have been identified (Rizvi and Gores, 2013; Rizvi et al., 2018). Several hormones and growth factors promote proliferation, exert effects and regulate biliary proliferation in CCA (Banales et al., 2016).

Gastrin peptides and their receptors potentiate the progression of gastrointestinal malignancies in the presence of inflammation (Aly et al., 2004). Hypergastrinemia is most concerned mechanism for possible carcinogenesis in PPI users, and defined as serum gastrin levels above the normal range (>150 pg./mL). PPIs use induced persistent elevation in antral pH and may be major cause of chronic hypergastrinemia, and is thought to stimulate cell proliferation and results in carcinogenesis and PPIs use is one of the major causes of hypergastrinemia, which may link PPIs use and tumor growth (Orlando et al., 2007). An immunochemical staining study demonstrated that gastrin precursor as well as receptor are overexpressed in CCA (Caplin et al., 1999).

Recent study demonstrated that PPIs use is associated with peri-ampullary tumor (Chien et al., 2016). It is reasonable to postulate that PPIs use and CCA are closely intercorrelated. There is a lack of data showing an association of PPIs use and CCA odds. To address this, we conducted a nationwide nested case-control study to analyze the odds of CCA among patients with PPIs use in Taiwan.

## Materials and methods

### Data source

The universal, single-payer National Health Insurance (NHI) program in Taiwan was initiated in 1995 and offers comprehensive medical coverage for all residents (Shen et al., 2014). The National Health Research Institute (NHRI) is in charge of the entire insurance claims database, namely, the NHIRD, which contains registration files and original medical claims data of all beneficiaries with encrypted unique personal identification numbers to ensure patient privacy. The NHIRD has been used extensively in many epidemiologic studies in Taiwan (Shen et al., 2014; Peng et al., 2015). Registry of Catastrophic Illness Database (RCIPD), a subset of the NHIRD, were defined as the case group. RCIPD is comprises data from insured residents with severe diseases, which are defined by the NHI program, such as malignancies, transplant, or autoimmune diseases, who are eligible to apply for the catastrophic illness certificate and are exempt from co-payments for NHI services. The diagnostic codes used in NHIRD are based on the International Classification of Diseases, 9th Revision, Clinical Modification (ICD-9-CM).

### Ethics statement

The NHIRD encrypts patient personal information to protect privacy and provides researchers with anonymous identification numbers associated with relevant claims information, including sex, date of birth, medical services received, and prescriptions. Therefore, patient consent is not required to access the NHIRD. This study was approved to fulfill the condition for exemption by the Institutional Review Board (IRB) of China Medical University (CMUH-104-REC2-115-CR2). The IRB also specifically waived the consent requirement.

### Study subjects

**Figure 1:**
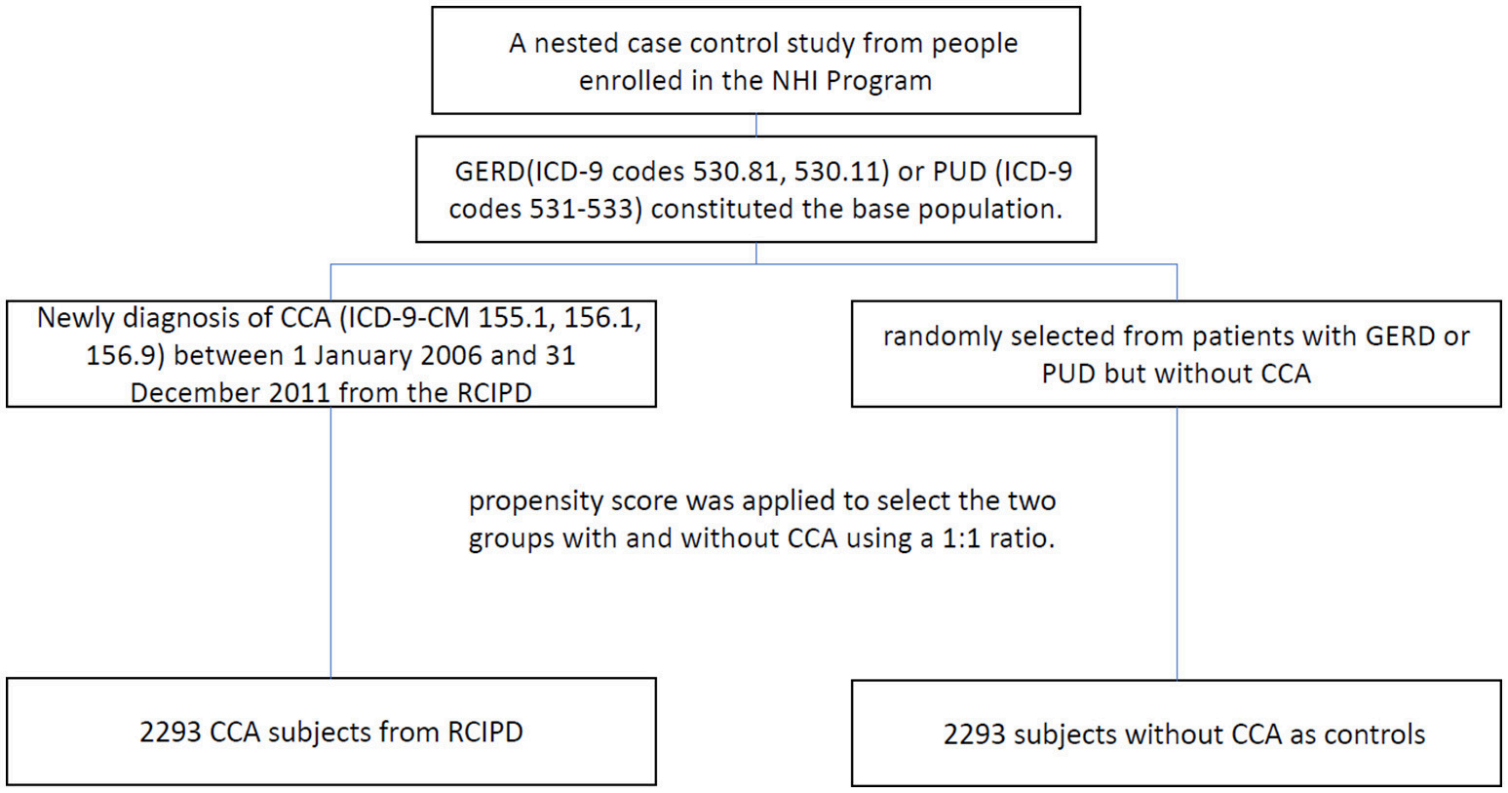
Study flow diagram (NHI, national health insurance; RCIPD, Registry of Catastrophic Illness Database; GERD, gastroesophageal reflux disease; PUD, peptic ulcer disease; CCA, cholangiocarcinoma.

Patients with gastroesophageal reflux disease (ICD-9 codes 530.81, 530.11) or peptic ulcer diseases (ICD-9 codes 531-533) constituted the base population. Patients with new diagnosis of CCA (ICD-9-CM 155.1, 156.1, 156.9) between 1 January 2006 and 31 December 2011 from the RCIPD. The date of diagnosis of CCA was defined as the index date. Patients were excluded if they had another malignancy (ICD-9-CM codes 140-208) before the index date, were younger than 20 years of age, or had missing information with respect to age or sex. Control subjects in the non-CCA group were randomly selected from patients with gastroesophageal reflux disease or peptic ulcer disease but without CCA. To reduce selection bias, propensity score was applied to select the two groups with and without CCA using a 1:1 ratio. The propensity score was calculated using logistic regression to estimate the probability of the CCA assignment based on the baseline variables including gender, age, year of diagnosis CCA, medications [H2RA (H2-receptor antagonist), aspirin, metformin] and comorbidities [gastric polyp, gastritis, cirrhosis, diabetes, chronic pancreatitis, hepatitis B infection, hepatitis C infection, inflammatory bowel disease, biliary tract disease, stroke, coronary arterial disease (CAD), chronic obstructive pulmonary disease (COPD), alcohol-related illness, Clonorchis and opisthorchis, helicobacterPylori]. Oral steroid was also considered in the data analysis. Finally, a total of 2293 cases with CCA and 2293 controls without CCA were included in this study (Figure 1).

### Dependent and covariates used in the model

We consider several well-known associated factors (covariates) of CCA (dependent) including age, sex, and medication of H2RA, aspirin, and metformin, and comorbidities of gastric polyp, gastritis, cirrhosis, diabetes, chronic pancreatitis, hepatitis B infection, hepatitis C infection, inflammatory bowel disease, biliary tract disease, stroke, coronary arterial disease (CAD), chronic obstructive pulmonary disease (COPD), alcohol-related illness, and clonorchis and opisthorchis. These chosen age, sex, medication, and comorbidities can help to clarify the independent influence of PPI on CCA odds.

### Exposure to proton pump inhibitors medication

Patients with claims for omeprazole (ATC A02BC01), pantoprazole (ATC A02BC02), lansoprazole (ATC A02BC03), rabeprazole (ATC A02BC04), and esomeprazole (ATC A02BC05) before the index date were classified as PPIs medication exposure. We calculated cumulative defined daily dose (DDD) of each type of PPI prescribed for the CCA case group and control group. The DDD was defined using the ATC/DDD system of the WHO collaborating center for drug statistics and methodology, was used as a unit for measuring a prescribed amount of a given drug; it was the assumed average maintenance dose per day of a drug consumed for its main indication in adults (WHO International Working Group for Drug Statistics Methodology, 2003). All PPIs were compared based on the same standard using the following formula: total amount of drug/amount of drug in a DDD = number of DDDs. Cumulative DDD was estimated as the sum of the dispensed DDDs of any PPIs. The overall cumulative duration of PPI was also calculated by summation of the daily supply of each type of PPI before the index date.

### Sensitivity analysis

We excluded patients with first time PPIs use with diagnosis CCA during less than or equal to 6 months. In order to avoid the observation period difference cause the any bias. Each patient only could be retrospectively observed for 5 years. To reduce selection bias, propensity score was applied to select the two groups with and without CCA according to all variables in the Table 1 (including gender, age, year of diagnosis CCA, medications [H2RA (H2-receptor antagonist), aspirin, metformin, oral steroid] and comorbidities [gastric polyp, gastritis, cirrhosis, diabetes, chronic pancreatitis, hepatitis B infection, hepatitis C infection, inflammatory bowel disease, biliary tract disease, stroke, coronary arterial disease (CAD), chronic obstructive pulmonary disease (COPD), alcohol-related illness, *Clonorchis* and *Opisthorchis, Helicobacterpylori*]) using a 1:1 ratio (Supplementary Table 1).

**Table 1:** Baseline characteristics of cholangiocarcinoma group and non-cholangiocarcinoma group.

	**Cholangiocarcinoma**				
	**No** ***N*** = **2,293**		**Yes** ***N*** = **2,293**		
	***N***	**%**	***n***	**%**	*p*-value^*^
**Gender**					0.62
Women	1,136	49.5	1,153	50.3	
Men	1,157	50.5	1,140	49.7	
**Age (year)**					0.85
Mean (SD)^*^	68.3	13.8	67.3	10.9	0.01
**MEDICATIONS**					
PPI	1,246	54.1	1,780	77.2	< 0.001
H2RA	2,011	87.9	1,996	87.1	0.50
Aspirin	1,161	50.6	1,171	51.1	0.77
Metformin	588	25.6	584	25.5	0.89
**BASELINE CO-MORBIDITIES**					
Gastric polyp	20	0.87	21	0.92	0.88
Gastritis	1,540	67.2	1,535	66.9	0.88
Cirrhosis	1,502	65.5	1,452	63.3	0.12
Diabetes	616	26.9	593	25.9	0.44
Chronic pancreatitis	28	1.22	29	1.26	0.89
Hepatitis B infection	366	16.0	365	15.9	0.97
Hepatitis C infection	258	11.3	276	12.0	0.41
Inflammatory bowel disease	90	3.92	83	3.62	0.59
Biliary tract disease	980	42.7	1,046	45.6	0.05
Stroke	282	12.3	260	11.3	0.31
CAD	835	36.4	797	34.8	0.24
COPD	1,064	46.4	991	43.2	0.03
Alcohol-related illness	252	11.0	241	10.5	0.60
Clonorchis and Opisthorchis	2	0.09	4	0.17	0.41
*Helicabacter pylori*	53	2.31	0	0.00	–

Chi-square test and

* *t-test comparing subjects with and without cholangiocarcinoma. Data are presented as the number of subjects in each group, with percentages given in parentheses*.

### Statistical analysis

The Chi-square test was used to examine the differences in categorical variables between the CCA and non-CCA groups, while the two sample *t*-test was used to examine continuous variables. Univariable and multivariable logistic regression were used to estimate the effect of medication treatment and comorbidities on the Odds of CCA as indicated by the odds ratio (OR) with 95% confidence interval (CI). All analyses were performed using SAS statistical software (version 9.4; SAS Institute, Inc., Cary, NC), and results were considered statistically significant when two-tailed *p*-values were less than 0.05.

## Results

### Demographics and characteristics of study subjects

Table 1 shows that the two study groups were similar in distributions of gender, age, medications (H2RA, aspirin, and metformin), and comorbidities. The mean age of the CCA cases and non-CCA controls were 67.3 (±10.9) and 68.3 (±13.8) years. Patients with CCA tended to have a higher prevalence of PPI use, and oral steroiduse than subjects in the non-CCA group (*p*-values < 0.001).

### Odds of CCA associated with proton pump inhibitor and covariates

Table 2 shows the ORs of estimated CCA odds based on PPI use. Use of a PPI was associated with a significantly increased association of CCA [adjusted OR (aOR) = 2.57, 95% CI = 2.24–2.94]. Compared with patients aged ≥75 years, those who were ≤ 64 years and 65–74 years had higher odds for CCA, respectively (aOR = 1.32, 95% CI = 1.14–1.54; aOR = 1.60, 95% CI = 1.36–1.87).

**Table 2:** Odds ratios and 95% confidence intervals of cholangiocarcinoma associated with proton pump inhibitor and covariates.

	**Crude**		**Adjusted**^†^	
**Variable**	**OR**	**(95% CI)**	**OR**	**(95% CI)**
**GENDER**				
Women	1	(Reference)	1	(Reference)
Men	0.97	(0.87, 1.09)	–	–
**AGE, YEARS**				
≤ 64	1.31	(1.14, 1.50)^***^	1.32	(1.14, 1.54)^***^
65–74	1.61	(1.39, 1.87)^***^	1.60	(1.36, 1.87)^***^
≥75	1	(Reference)	1	(Reference)
**MEDICATIONS**				
PPI	2.58	(2.27, 2.93)^***^	2.57	(2.24, 2.94)^***^
H2RA	0.94	(0.79, 1.12)	–	–
Aspirin	1.02	(0.91, 1.14)	–	–
Metformin	0.99	(0.87, 1.13)	–	–
**BASELINE CO-MORBIDITIES**				
Gastric polyp	1.05	(0.57, 1.94)	–	–
**Gastritis**	0.99	(0.88, 1.12)	–	–
Cirrhosis	0.91	(0.81, 1.03)	–	–
Diabetes	0.95	(0.83, 1.08)	–	–
Chronic pancreatitis	1.04	(0.61, 1.75)	–	–
Hepatitis B infection	1.00	(0.85, 1.17)	–	–
Hepatitis C infection	1.08	(0.90, 1.29)	–	–
Inflammatory bowel disease	0.92	(0.68, 1.25)	–	–
Biliary tract disease	1.12	(1.00, 1.26)^*^	1.02	(0.90, 1.15)
Stroke	0.91	(0.76, 1.09)	–	–
CAD	0.93	(0.82, 1.05)	–	–
COPD	0.88	(0.78, 1.00)	0.73	(0.64, 0.83)^***^
Alcohol-related illness	0.95	(0.79, 1.15)	–	–
Clonorchis and Opisthorchis	2.00	(0.37, 10.9)	–	–
*Helicabacter pylori*	–	–	–	–

† Adjusted for age group, oral steroid, biliary tract disease, and COPD;

* p < 0.05;

*** *p < 0.001*.

### Dosage of proton pump inhibitors and odds of CCA

For individual PPIs, CCA odds were the highest in patients using < 110 cumulative DDD of esomeprazole (aOR = 5.16, 95% CI = 3.98–6.69), followed by < 70 cumulative DDD of pantoprazole (aOR = 4.42, 95% CI = 3.45–5.65), < 110 cumulative DDD of rabeprazole (aOR = 4.14, 95% CI = 2.82–6.07), < 145 cumulative DDD of lansoprazole (aOR = 3.49, 95% CI = 2.84–4.30), and < 145 cumulative DDD of omeprazole (aOR = 1.64, 95% CI = 1.36–1.98) (Table 3).

**Table 3:** Odds ratio and 95% confidence intervals of cholangiocarcinoma associated with cumulative DDD dose of individual proton pump inhibitors.

	**Case number/control number**	**Crude odds ratio**	**(95% CI)**	**Adjusted odds ratio**^†^	**(95% CI)**	**Adjusted odds ratio(95% CI)^†^**
Non-use of PPI	523/991	1.00	(reference)	1.00	(reference)	
**OMEPRAZOLE**^#^						
< 80 DDD	363/409	1.68	(1.41, 2.01)^***^	1.64	(1.36, 1.98)^***^	1.00 (reference)
≥80 DDD	101/151	1.27	(0.96, 1.67)	1.16	(0.86, 1.56)	0.72(0.53, 0.99)^*^
p for trend		< 0.001		< 0.001		
**PANTOPRAZOLE**^#^						
< 70 DDD	285/122	4.43	(3.49, 5.61)^***^	4.42	(3.45, 5.65)^***^	1.00 (reference)
≥70 DDD	55/66	1.58	(1.09, 2.29)^***^	1.52	(1.02, 2.26)^*^	0.35 (0.22, 0.53)^***^
p for trend		< 0.001		< 0.001		
**LANSOPRAZOLE**^#^						
< 145 DDD	393/208	3.58	(2.94, 4.37)^***^	3.49	(2.84, 4.30)^***^	1.00 (reference)
≥145 DDD	94/111	1.61	(1.20, 2.15)^***^	1.65	(1.21, 2.24)^**^	0.47 (0.33, 0.66)^***^
p for trend		< 0.001		< 0.001		
**RABEPRAZOLE**^#^						
< 110 DDD	99/44	4.26	(2.94, 6.18)^***^	4.14	(2.82, 6.07)^***^	1.00 (reference)
≥110 DDD	30/17	3.34	(1.83, 6.12)^***^	3.18	(1.70, 5.98)^***^	0.76 (0.37, 1.55)
p for trend		< 0.001		< 0.001		
**ESOMEPRAZOLE**^#^						
< 110 DDD	286/102	5.31	(4.14, 6.82)^***^	5.16	(3.98, 6.69)^***^	1.00 (reference)
≥110 DDD	64/72	1.68	(1.18, 2.40)^***^	1.66	(1.14, 2.41)^**^	0.33 (0.21, 0.50)^***^
p for trend		< 0.001		< 0.001		

# The cumulative DDD dose is partitioned into 2 segments by third quartile.

† Adjusted for age group, oral steroid, biliary tract disease, and COPD;

*p < 0.05,

**p < 0.01,

***p *< 0.001*.

### PPIs use and odds of intrahepatic and extrahepatic CCA

Table 4 shows the CCA odds by different locations and PPIs use. Compared with the non-CCA group, PPI users had a greater association with extrahepatic cancer (aOR = 2.41, 95% CI = 1.82–3.18), intrahepatic cancer (aOR = 1.84, 95% CI = 1.59–2.14), and unspecified CCA cancer (aOR = 1.51, 95% CI = 1.22–1.86), respectively.

**Table 4:** Odds ratios and 95% confidence intervalsin various subtypes of cholangiocarcinoma associated with proton pump inhibitor.

**Variable**	***N***	**Crude odds ratio (95% CI)**	**Adjusted odds ratio^†^ (95% CI)**
**CHOLANGIOCARCINOMA**			
None	2,293	1 (Reference)	1 (Reference)
Intrahepatic	1,381	1.56 (1.27, 1.92)^***^	1.84 (1.59, 2.14)^***^
Extrahepatic	373	2.53 (1.92, 3.34)^***^	2.41 (1.82, 3.18)^***^
Unspecified	539	1.56 (1.27, 1.92)^***^	1.51 (1.22, 1.86)^***^

† Adjusted for age group, oral steroid, biliary tract disease, and COPD;

***p *< 0.001*.

## Discussion

In general, our study demonstrated that PPIs use is associated with all types of CCA, with an adjusted OR of 2.57, and adjusted OR for intrahepatic and extrahepatic CCA of 1.92 and 2.51, respectively. In our results, PPIs use was associated with odds of intrahepatic and extrahepatic CCA.

It has been established that the pleotropic effect of PPIs *in vivo* (Yoshida et al., 2000; Simon et al., 2006; Namazi and Jowkar, 2008). By epidemiological studies, the use of PPIs and associated of cancer is still questionable. Gastric cancer is mostly investigated, and is still variable (Poulsen et al., 2009; Song et al., 2014; Attwood et al., 2015). A recent report demonstrated long-term PPIs use is associated with gastric cancer despite of *Helicobacter pylori* eradication (Cheung et al., 2018). For the pancreas and hepatobiliary system, previous results demonstrated that neither H2RA nor PPIs was associated with pancreatic cancer (Bradley et al., 2012). Previous findings in a human study that demonstrated hepatocellular carcinoma and CCA express the CCK-B/gastric receptor and precursor forms of gastrin. This expression may be associated with tumor proliferation (Caplin et al., 1999). PPI is reported to be is associated with a dose-dependent association of progression of chronic liver disease to cirrhosis, as well as an increased association of hepatic decompensation and hepatocellular carcinoma (Li et al., 2018). PPIs use increased the odds of peri-ampulla cancer is reported mild odds with OR 1.35 (Chien et al., 2016). Data on PPIs use and odds of CCA are still lacking. In the present study, our results demonstrated odds of PPIs use and intrahepatic and extrahepatic CCA, and the odds seemed to be about 2-folds.

In the era of PPIs use, concern about the potential development of chronic hypergastrinemia has risen because of the widespread use of PPIs for acid-related disorders. In addition to its stimulating effect on gastric acid secretion, gastrin may act as a potent cell-growth factor in a variety of normal and abnormal biological processes including maintenance of the gastric mucosa, proliferation of ECL cells, and neoplastic transformation (Rozengurt and Walsh, 2001; Chao and Hellmich, 2010).

Recently, an increased association of cancer for PPI use is growing reported (Cheung et al., 2018; Li et al., 2018). We first reported the association of CCA and PPI use. There were several limitations in this study. First, the patients' compliance with medication could not be confirmed. We presumed that all prescribed PPIs were taken regularly by all patients enrolled. Second, Metformin, H2RA, and aspirin were adjusted for in our study, but the interaction of other medications with PPIs were not considered. We also assumed that the medication conditions were homogenous in both groups. Third, patients' lifestyle, alcohol, and smoking, which are important factors associated with cancer, were not assessed as these data are not collected in the NHIRD. Fourth, it is possible that variations and mistakes existed in the registry of diagnosis, which could potentially have influenced the results. Fifth, the majority of patients may have taken more than one type of PPIs and the interaction of different PPIs could not be completely accounted for. Sixth, the duration of exposure to PPIs can have an impact on the odds of CCA. Though cumulative DDDs could be related to the duration, but the duration of PPIs use was not well defined in the present study. Furthermore, it was assumed that over-the-counter PPIs comprised only a small portion of all PPIs, but the exact proportion was not evaluated.

There are odds of intrahepatic and extrahepatic CCA among PPIs users but not in dose-dependent models. All PPIs use was associated with odds of CCA. Physicians description as well as PPI users would take care odds of CCA. Also, the conclusion of this study applies to a well-defined homogeneous population that may not be directly extrapolated for example to Caucasian. Analyses of larger numbers of cases and further investigations for validate the use of PPI and cancer odds is needed.

## Author contributions

Y-CP, C-LL, C-HK: conception and design; C-HK: administrative support; All authors: collection and assembly of data, data analysis and interpretation, manuscript writing, and final approval of manuscript.

### Conflict of interest statement

The authors declare that the research was conducted in the absence of any commercial or financial relationships that could be construed as a potential conflict of interest.
