# Supplementary material for: Association Between Cholangiocarcinoma and Proton Pump Inhibitors Use: A Nested Case-Control Study
Source: Front Pharmacol. 2018 Jul 3;9:718. doi: 10.3389/fphar.2018.00718 (PMC6037835; doi:10.3389/fphar.2018.00718)
Supplement: Supplementary file 1 [file Table_1.pdf]

Supplementary table 1 Odds ratios and 95% confidence intervals in various subtypes of cholangiocarcinoma associated with proton pump inhibitor for sensitivity analysis

Variable	Case number/ control number N=1348/1348	Crude odds ratio (95% CI)
Cholangiocarcinoma		
None	881/938	1(Reference)
Intrahepatic	278/599	1.07(0.90, 1.28)
Extrahepatic	80/797	1.55(1.15, 2.08)**
Unspecified	109/768	1.03(0.80, 1.31)
CCA	467/410	1.21(1.03, 1.43)*

p<0.05; **p<0.01;
